# Supplementary material for: Rapid Analysis of Aristolochic Acids and Aristolactams in Houttuyniae Herba by LC–MS/MS
Source: Molecules. 2022 Dec 16;27(24):8969. doi: 10.3390/molecules27248969 (PMC9788189; doi:10.3390/molecules27248969)
Supplement: Supplementary file 1 [file molecules-27-08969-s001.zip › molecules-2086028-supplementary.pdf]

molecules

MDPI

**Table S1.** Results of recovery.

| | Sampling amount(g) | Sample content(μg) | Added amount(μg) | Detected amount(μg) | Recovery(%) | Average recovery(%) | RSD(%) |
|---|---|---|---|---|---|---|---|
| | 0.2538 | 0.60 | | 0.963 | 85 | | |
| | 0.2523 | 0.60 | 0.42 | 0.955 | 84 | 84 | 4.4 |
| | 0.2544 | 0.60 | | 0.946 | 81 | | |
| | 0.2536 | 0.60 | | 1.04 | 84 | | |
| C1 | 0.2537 | 0.60 | 0.53 | 1.06 | 87 | 85 | 2.9 |
| | 0.2530 | 0.60 | | 1.04 | 83 | | |
| | 0.2537 | 0.60 | | 1.18 | 91 | | |
| | 0.2539 | 0.60 | 0.63 | 1.13 | 84 | 89 | 4.6 |
| | 0.2537 | 0.60 | | 1.18 | 92 | | |
| | 0.2538 | 1.8 | | 3.18 | 85 | | |
| | 0.2523 | 1.8 | 1.0 | 3.20 | 87 | 87 | 3.5 |
| | 0.2544 | 1.8 | | 3.26 | 90 | | |
| | 0.2536 | 1.9 | | 3.47 | 83 | | |
| C2 | 0.2537 | 1.9 | 1.9 | 3.55 | 87 | 84 | 3.4 |
| | 0.2530 | 1.9 | | 3.44 | 82 | | |
| | 0.2537 | 1.9 | | 3.88 | 87 | | |
| | 0.2539 | 1.9 | 2.3 | 3.89 | 87 | 87 | 3.2 |
| | 0.2537 | 1.9 | | 3.89 | 87 | | |
| | 0.2538 | 0.00 | | 18.1 | 114 | | |
| | 0.2523 | 0.00 | 16.0 | 18.1 | 113 | 111 | 5.1 |
| | 0.2544 | 0.00 | | 17.0 | 106 | | |
| | 0.2536 | 0.00 | | 23.3 | 117 | | |
| C3 | 0.2537 | 0.00 | 20.0 | 24.2 | 121 | 119 | 3.8 |
| | 0.2530 | 0.00 | | 23.9 | 120 | | |
| | 0.2537 | 0.00 | | 28.8 | 120 | | |
| | 0.2539 | 0.00 | 24.0 | 28.2 | 118 | 118 | 4.6 |
| | 0.2537 | 0.00 | | 27.9 | 116 | | |
| | 0.2538 | 0.00 | | 12.0 | 111 | | |
| | 0.2523 | 0.00 | 10.7 | 11.0 | 103 | 108 | 5.8 |
| | 0.2544 | 0.00 | | 11.9 | 111 | | |
| | 0.2536 | 0.00 | | 15.8 | 118 | | |
| C4 | 0.2537 | 0.00 | 13.4 | 15.6 | 116 | 118 | 3.0 |
| | 0.2530 | 0.00 | | 16.0 | 119 | | |
| | 0.2537 | 0.00 | | 18.1 | 113 | | |
| | 0.2539 | 0.00 | 16.1 | 17.9 | 111 | 111 | 4.5 |
| | 0.2537 | 0.00 | | 17.5 | 109 | | |
| | 0.2538 | 2.5 | | 4.48 | 102 | | |
| | 0.2523 | 2.5 | 2.0 | 4.45 | 101 | 103 | 3.0 |
| | 0.2544 | 2.5 | | 4.58 | 107 | | |
| | 0.2536 | 2.5 | | 5.06 | 106 | | |
| C5 | 0.2537 | 2.5 | 2.4 | 5.19 | 111 | 107 | 3.4 |
| | 0.2530 | 2.5 | | 5.03 | 104 | | |
| | 0.2537 | 2.5 | | 5.54 | 104 | | |
| | 0.2539 | 2.5 | 2.9 | 5.50 | 103 | 104 | 1.8 |
| | 0.2537 | 2.5 | | 5.54 | 105 | | |
| C6 | 0.2538 | 0.00 | 10.7 | 13.1 | 123 | 119 | 3.5 |

| | | | | | | | |
|---|---|---|---|---|---|---|---|
| | 0.2523 | 0.00 | | 12.3 | 115 | | |
| | 0.2544 | 0.00 | | 12.7 | 118 | | |
| | 0.2536 | 0.00 | | 16.1 | 120 | | |
| | 0.2537 | 0.00 | 13.4 | 16.0 | 119 | 120 | 1.6 |
| | 0.2530 | 0.00 | | 16.1 | 120 | | |
| | 0.2537 | 0.00 | | 19.4 | 121 | | |
| | 0.2539 | 0.00 | 16.1 | 19.4 | 121 | 119 | 1.8 |
| | 0.2537 | 0.00 | | 18.9 | 117 | | |
| | 0.2538 | 1.6 | | 2.64 | 74 | | |
| | 0.2523 | 1.6 | 1.3 | 2.67 | 77 | 77 | 4.4 |
| | 0.2544 | 1.6 | | 2.72 | 80 | | |
| | 0.2536 | 1.6 | | 2.96 | 78 | | |
| C7 | 0.2537 | 1.6 | 1.7 | 3.00 | 81 | 79 | 2.9 |
| | 0.2530 | 1.6 | | 2.95 | 79 | | |
| | 0.2537 | 1.6 | | 3.21 | 78 | | |
| | 0.2539 | 1.6 | 2.0 | 3.33 | 84 | 81 | 4.3 |
| | 0.2537 | 1.6 | | 3.29 | 82 | | |
| | 0.2538 | 0.00 | | 44.3 | 112 | | |
| | 0.2523 | 0.00 | 39.4 | 44.5 | 113 | 113 | 1.6 |
| | 0.2544 | 0.00 | | 44.4 | 113 | | |
| | 0.2536 | 0.00 | | 52.9 | 107 | | |
| C8 | 0.2537 | 0.00 | 49.3 | 53.7 | 109 | 109 | 2.2 |
| | 0.2530 | 0.00 | | 55.2 | 112 | | |
| | 0.2537 | 0.00 | | 63.1 | 107 | | |
| | 0.2539 | 0.00 | 59.2 | 68.0 | 115 | 112 | 4.0 |
| | 0.2537 | 0.00 | | 68.2 | 115 | | |
| | 0.2538 | 8.4 | | 14.6 | 97 | | |
| | 0.2523 | 8.3 | 6.4 | 14.4 | 95 | 98 | 3.8 |
| | 0.2544 | 8.4 | | 14.9 | 101 | | |
| | 0.2536 | 8.3 | | 17.0 | 108 | | |
| C9 | 0.2537 | 8.4 | 8.0 | 17.5 | 115 | 108 | 5.0 |
| | 0.2530 | 8.3 | | 16.5 | 102 | | |
| | 0.2537 | 8.4 | | 18.7 | 108 | | |
| | 0.2539 | 8.4 | 9.6 | 18.3 | 103 | 104 | 3.7 |
| | 0.2537 | 8.4 | | 18.0 | 101 | | |
| | 0.2538 | 0.00 | | 1.93 | 116 | | |
| | 0.2523 | 0.00 | 1.7 | 1.94 | 117 | 118 | 2.3 |
| | 0.2544 | 0.00 | | 1.99 | 120 | | |
| | 0.2536 | 0.00 | | 2.52 | 121 | | |
| C10 | 0.2537 | 0.00 | 2.1 | 2.49 | 120 | 119 | 3.2 |
| | 0.2530 | 0.00 | | 2.42 | 117 | | |
| | 0.2537 | 0.00 | | 2.84 | 114 | | |
| | 0.2539 | 0.00 | 2.5 | 2.81 | 113 | 112 | 2.5 |
| | 0.2537 | 0.00 | | 2.73 | 110 | | |
| | 0.2538 | 4.5 | | 8.38 | 105 | | |
| | 0.2523 | 4.5 | 3.6 | 8.14 | 99 | 104 | 4.5 |
| | 0.2544 | 4.5 | | 8.49 | 108 | | |
| C11 | 0.2536 | 4.5 | | 9.47 | 108 | | |
| | 0.2537 | 4.5 | 4.6 | 9.58 | 111 | 107 | 4.1 |
| | 0.2530 | 4.5 | | 9.13 | 101 | | |
| | 0.2537 | 4.5 | 5.5 | 10.5 | 108 | 108 | 3.8 |

| | | | | | | | |
|---|---|---|---|---|---|---|---|
| | 0.2539 | 4.5 | | 10.2 | 103 | | |
| | 0.2537 | 4.5 | | 10.6 | 111 | | |
| | 0.2538 | 0.00 | | 11.6 | 113 | | |
| | 0.2523 | 0.00 | 10.3 | 11.5 | 112 | 113 | 1.5 |
| | 0.2544 | 0.00 | | 11.6 | 113 | | |
| | 0.2536 | 0.00 | | 14.6 | 114 | | |
| C12 | 0.2537 | 0.00 | 12.8 | 15.4 | 120 | 118 | 3.9 |
| | 0.2530 | 0.00 | | 15.5 | 121 | | |
| | 0.2537 | 0.00 | | 18.4 | 119 | | |
| | 0.2539 | 0.00 | 15.4 | 18.5 | 120 | 120 | 1.0 |
| | 0.2537 | 0.00 | | 18.5 | 120 | | |

**Table S2.** Contents of six alkaloids in dried Houttuyniae herba ($\bar{x} \pm s$,n=3, μg/g).

| | C1 | C2 | C5 | C7 | C9 | C11 | Total |
|---|---|---|---|---|---|---|---|
| S1 | 0.55±0.01 | 5.1±0.06 | 7.3±0.2 | 6.9±0.2 | 8.8±0.2 | 10.1±0.1 | 39 |
| S2 | 2.1±0.02 | 3.8±0.04 | 21.7±0.5 | 11.5±0.06 | 58.2±0.6 | 34.4±0.2 | 130 |
| S3 | 0.067±0.002 | 2.1±0.1 | 5.3±0.07 | 6.8±0.06 | 23.0±0.2 | 5.5±0.4 | 43 |
| S4 | 2.0±0.03 | 6.1±0.1 | 28.2±1.0 | 21.5±0.4 | 54.7±0.2 | 32.1±0.1 | 145 |
| S5 | 0.032±0.001 | 2.3±0.006 | 3.1±0.01 | 11.9±0.04 | 14.6±0.3 | 3.4±0.06 | 35 |
| S6 | 1.7±0.03 | 7.1±0.1 | 5.0±0.1 | 10.5±0.05 | 19.5±0.5 | 5.7±0.1 | 49 |
| S7 | 1.5±0.02 | 4.4±0.05 | 3.4±0.03 | 6.2±0.1 | 9.0±0.1 | 11.8±0.2 | 36 |
| S8 | 9.1±0.09 | 16.3±0.2 | 8.9±0.3 | 11.6±0.2 | 46.7±0.4 | 26.9±0.2 | 119 |
| S9 | 1.3±0.02 | 5.0±0.03 | 5.3±0.08 | 10.4±0.07 | 13.5±0.3 | 8.3±0.1 | 44 |
| S10 | 2.1±0.04 | 6.3±0.03 | 8.4±0.1 | 4.2±0.1 | 15.7±0.4 | 16.9±0.4 | 54 |
| S11 | 13.5±0.06 | 10.9±0.07 | 42.9±0.06 | 28.1±0.6 | 75.2±0.9 | 162.2±0.008 | 333 |
| S12 | 0.55±0.006 | 3.4±0.04 | 7.6±0.1 | 11.8±0.2 | 19.3±0.4 | 12.1±0.04 | 55 |
| S13 | 2.0±0.04 | 7.9±0.04 | 72.4±0.3 | 58.0±0.08 | 198.9±2.2 | 192.1±0.2 | 531 |
| S14 | 2.4±0.08 | 7.6±0.2 | 10.3±0.2 | 7.1±0.03 | 33.9±0.4 | 17.9±0.3 | 79 |
| S15 | 7.2±0.1 | 12.6±0.06 | 19.7±0.1 | 5.8±0.06 | 60.1±0.6 | 34.5±0.6 | 140 |
| S17 | 0.27±0.0 | 3.0±0.01 | 19.7±0.5 | 19.9±0.4 | 89.1±1.0 | 21.2±0.3 | 153 |
| S18 | 0.18±0.003 | 27.5±0.4 | 43.6±0.009 | 177.3±0.1 | 147.2±2.7 | 38.3±0.5 | 434 |
| S19 | 2.8±0.05 | 11.1±0.1 | 2.6±0.03 | 16.8±0.2 | 11.1±0.2 | 55.9±0.6 | 100 |
| S20 | 0.029±0.001 | 2.1±0.08 | 5.7±0.2 | 9.7±0.3 | 11.3±0.3 | 2.0±0.03 | 31 |
| S32 | 0.78±0.007 | 3.4±0.05 | 2.9±0.04 | 1.7±0.08 | 3.5±0.04 | 5.3±0.07 | 18 |
| S33 | 6.2±0.7 | 10.3±0.1 | 32.8±0.6 | 10.4±0.2 | 116.6±1.0 | 76.9±0.4 | 253 |
| S34 | 0.078±0.001 | 2.7±0.1 | 9.5±0.6 | 9.5±0.3 | 32.2±0.1 | 1.6±0.01 | 56 |
| S35 | 6.81±0.009 | 9.8±0.1 | 39.1±0.0 | 20.5±0.1 | 100.6±0.7 | 128.8±0.4 | 306 |
| S36 | 0.034±0.001 | 1.7±0.01 | 1.3±0.02 | 2.1±0.008 | 5.9±0.04 | 1.1±0.001 | 12 |
| S37 | 4.7±0.02 | 8.7±0.05 | 4.7±0.06 | 6.0±0.09 | 30.5±0.08 | 19.0±0.2 | 74 |
| S38 | 4.4±0.2 | 8.7±0.04 | 4.7±0.1 | 6.0±0.03 | 30.7±0.5 | 18.8±0.2 | 73 |
| S39 | 12.9±0.1 | 9.7±0.8 | 6.1±0.2 | 10.6±0.6 | 33.4±0.9 | 40.0±0.3 | 113 |
| S40 | 0.91±0.01 | 3.8±0.04 | 2.1±0.03 | 3.2±0.09 | 9.0±0.1 | 2.8±0.02 | 22 |
| S41 | 1.4±0.01 | 5.2±0.08 | 8.4±0.1 | 4.8±0.08 | 24.1±0.6 | 15.5±0.2 | 59 |
| S42 | 26.4±0.2 | 17.5±0.2 | 45.5±0.3 | 24.9±0.1 | 94.7±0.07 | 184.0±0.4 | 393 |
| S43 | 0.92±0.005 | 3.0±0.02 | 4.8±0.07 | 8.9±0.06 | 22.7±0.4 | 14.8±0.06 | 55 |
| S44 | 1.9±0.02 | 6.9±0.1 | 46.3±0.03 | 34.2±0.4 | 178.5±0.4 | 189.6±0.2 | 457 |
| S45 | 11.8±0.05 | 9.7±0.1 | 11.4±0.03 | 7.2±0.1 | 49.9±0.3 | 60.6±0.5 | 151 |
| S46 | 12.0±0.02 | 14.7±0.1 | 19.6±0.2 | 5.7±0.2 | 64.8±0.7 | 35.5±0.3 | 152 |
| S47 | 0.3±0.001 | 28.5±0.3 | 74.4±0.4 | 321.8±0.2 | 350.7±0.9 | 98.6±1.2 | 874 |
| S48 | 0.4±0.004 | 3.1±0.01 | 13.3±0.2 | 16.4±0.07 | 98.9±0.5 | 35.0±0.4 | 167 |
| S49 | 5.2±0.08 | 16.1±0.1 | 3.0±0.06 | 29.4±0.5 | 26.7±0.4 | 131.6±0.4 | 212 |

| | | | | | | | |
|---|---|---|---|---|---|---|---|
| S50 | 0.096±0.001 | 3.0±0.02 | 5.1±0.04 | 7.3±2.3 | 11.7±0.2 | 2.3±0.03 | 30 |
| S54 | 0.39±0.006 | 6.1±0.05 | 17.4±0.2 | 7.9±3.0 | 10.1±0.08 | 3.2±0.1 | 45 |
| S55 | 0.20±0.002 | 1.1±0.01 | 14.0±0.3 | 6.9±0.07 | 22.5±0.3 | 5.2±0.05 | 50 |
| S56 | 0.042±0.0 | 3.7±0.02 | 6.1±0.2 | 8.3±0.04 | 15.1±0.07 | 0.88±0.008 | 34 |
| S57 | 0.58±0.005 | 2.8±0.02 | 16.1±0.3 | 12.2±0.3 | 18.1±0.2 | 20.9±0.1 | 71 |
| S58 | 0.018±0.0 | 1.0±0.01 | 2.9±0.03 | 10.1±0.1 | 8.2±0.3 | 2.0±0.02 | 24 |
| S59 | 0.32±0.001 | 6.0±0.04 | 12.3±0.5 | 15.2±0.2 | 13.0±0.4 | 0.64±0.002 | 47 |
| S60 | 0.58±0.006 | 5.2±0.06 | 3.0±0.07 | 3.3±0.1 | 2.8±0.004 | 1.9±0.02 | 17 |
| S61 | 0.84±0.007 | 3.9±0.05 | 3.6±0.02 | 4.2±0.1 | 6.4±0.3 | 3.3±0.02 | 22 |
| S62 | 0.25±0.004 | 1.6±0.04 | 2.7±0.02 | 4.3±0.1 | 2.6±0.04 | 0.72±0.001 | 12 |
| S63 | 0.083±0.001 | 1.3±0.01 | 6.0±0.08 | 3.6±0.03 | 6.0±0.02 | 2.5±0.01 | 19 |
| S64 | 1.8±0.02 | 4.2±0.06 | 27.4±0.8 | 26.3±0.1 | 29.9±0.5 | 92.7±0.2 | 182 |
| S65 | 0.17±0.0 | 4.0±0.02 | 11.6±0.4 | 24.6±0.2 | 16.6±0.2 | 2.8±0.2 | 60 |
| S66 | 0.24±0.001 | 4.1±0.04 | 22.7±0.5 | 29.8±0.5 | 41.9±0.5 | 33.5±0.4 | 132 |
| S67 | 0.49±0.002 | 4.1±0.09 | 4.9±0.03 | 5.5±0.009 | 9.1±0.2 | 4.3±0.08 | 28 |
| S68 | 0.72±0.01 | 3.9±0.04 | 11.5±0.2 | 3.7±0.04 | 8.6±0.1 | 2.6±0.01 | 31 |
| S69 | 0.13±0.001 | 21.8±0.2 | 38.8±0.05 | 160.4±0.1 | 124.5±1.4 | 34.3±0.7 | 380 |
| S70 | 0.13±0.003 | 3.0±0.1 | 28.8±2.2 | 36.9±0.02 | 89.0±0.6 | 6.2±0.2 | 164 |
| S71 | 0.24±0.004 | 2.9±0.03 | 1.4±0.01 | 9.5±0.04 | 2.5±0.04 | 9.3±0.3 | 26 |
| S72 | 0.012±0.0 | 1.8±0.020 | 4.6±0.07 | 9.1±0.2 | 5.8±0.1 | 0.25±0.0 | 22 |

**Table S3.** Contents of six alkaloids in fresh Houttuyniae herba ($\bar{x} \pm s$,n=3, μg/g).

| | C1 | C2 | C5 | C7 | C9 | C11 | Total |
|---|---|---|---|---|---|---|---|
| S16 | 0.11±0.002 | 0.44±0.004 | 2.9±0.07 | 2.0±0.008 | 0.31±0.02 | 0.74±0.001 | 7 |
| S21 | 3.6±0.007 | 8.1±0.3 | 16.9±0.5 | 27.0±0.6 | 16.3±0.4 | 27.9±0.1 | 100 |
| S22 | 1.1±0.004 | 7.4±0.05 | 17.3±0.06 | 25.9±0.4 | 12.6±0.1 | 23.1±0.1 | 87 |
| S23 | 2.6±0.04 | 7.8±0.02 | 17.7±0.3 | 27.8±0.2 | 14.5±0.3 | 28.2±0.2 | 99 |
| S24 | 0.21±0.004 | 1.9±0.01 | 6.2±0.2 | 4.8±0.03 | 2.8±0.05 | 0.40±0.0 | 16 |
| S25 | 0.073±0.001 | 1.3±0.006 | 5.1±0.3 | 5.0±0.04 | 2.4±0.02 | 2.5±0.03 | 16 |
| S26 | 0.11±0.001 | 3.1±0.04 | 4.9±0.1 | 6.5±0.1 | 2.8±0.02 | 1.6±0.02 | 19 |
| S27 | 2.4±0.02 | 9.5±0.2 | 45.1±0.1 | 112.6±0.1 | 35.0±0.7 | 62.0±0.7 | 267 |
| S28 | 3.0±0.04 | 8.5±0.1 | 47.1±0.02 | 123.0±0.1 | 45.8±0.7 | 95.4±0.3 | 323 |
| S29 | 2.0±0.02 | 8.4±0.1 | 50.1±0.009 | 144.4±0.2 | 38.2±0.2 | 56.5±0.5 | 300 |
| S30 | 2.3±0.04 | 10.4±0.1 | 53.3±0.04 | 154.6±0.7 | 45.4±0.7 | 66.3±0.4 | 332 |
| S31 | 2.6±0.01 | 9.1±0.1 | 46.6±0.05 | 121.0±0.2 | 38.6±0.7 | 74.8±0.3 | 293 |
| S51 | 1.5±0.008 | 3.1±0.07 | 4.3±0.04 | 9.0±0.08 | 7.5±0.2 | 17.6±0.06 | 43 |
| S52 | 1.5±0.03 | 2.9±0.006 | 4.4±0.05 | 8.3±0.3 | 7.1±0.3 | 17.0±0.3 | 41 |
| S53 | 2.7±0.05 | 3.5±0.03 | 4.1±0.02 | 7.8±0.1 | 6.3±0.02 | 16.9±0.2 | 41 |
| S73 | 1.1±0.01 | 10.3±0.2 | 54.3±0.09 | 110.0±0.4 | 47.4±0.5 | 42.4±0.4 | 266 |
| S74 | 1.7±0.02 | 11.9±0.1 | 33.3±0.2 | 53.6±0.9 | 18.2±0.1 | 22.3±0.3 | 141 |
| S75 | 2.0±0.009 | 13.7±0.1 | 33.8±0.07 | 51.6±0.2 | 19.6±0.2 | 25.2±0.1 | 146 |
